# Peptidomes and Structures Illustrate How SLA-I Micropolymorphism Influences the Preference of Binding Peptide Length

**DOI:** 10.3389/fimmu.2022.820881

**Published:** 2022-02-28

**Authors:** Xiaohui Wei, Shen Li, Suqiu Wang, Guojiao Feng, Xiaoli Xie, Zhuolin Li, Nianzhi Zhang

**Affiliations:** ^1^ Department of Microbiology and Immunology, College of Veterinary Medicine, China Agricultural University, Beijing, China; ^2^ National Health Commission (NHC) Key Laboratory of Human Disease Comparative Medicine, Beijing Key Laboratory for Animal Models of Emerging and Remerging Infectious Diseases, Institute of Laboratory Animal Science, Chinese Academy of Medical Sciences (CAMS) and Comparative Medicine Center, Peking Union Medical College, Beijing, China

**Keywords:** MHC class I, micropolymorphism, binding peptide length, crystal structure, peptidomes

## Abstract

Polymorphisms can affect MHC-I binding peptide length preferences, but the mechanism remains unclear. Using a random peptide library combined with LC-MS/MS and *de novo* sequencing (RPLD-MS) technique, we found that two swine MHC-I molecules with high sequence homology, SLA-1*04:01 and SLA-1*13:01, had significant differences in length preference of the binding peptides. Compared with SLA-1*04:01, SLA-1*13:01 binds fewer short peptides with 8-10 amino acids, but more long peptides. A dodecapeptide peptide (RW12) can bind to both SLA-1*04:01 and SLA-1*13:01, but their crystal structures indicate that the binding modes are significantly different: the entirety of RW12 is embedded in the peptide binding groove of SLA-1*04:01, but it obviously protrudes from the peptide binding groove of SLA-1*13:01. The structural comparative analysis showed that only five differential amino acids of SLA-1*13:01 and SLA-1*04:01 were involved in the binding of RW12, and they determine the different ways of long peptides binding, which makes SLA-1*04:01 more restrictive on long peptides than SLA-1*13:01, and thus binds fewer long peptides. In addition, we found that the N terminus of RW12 extends from the groove of SLA-1*13:01, which is similar to the case previously found in SLA-1*04:01. However, this unusual peptide binding does not affect their preferences of binding peptide length. Our study will be helpful to understand the effect of polymorphisms on the length distribution of MHC-I binding peptides, and to screen SLA-I-restricted epitopes of different lengths and to design effective epitope vaccines.

## Introduction

Highly polymorphic major histocompatibility complex class I (MHC-I) molecules present a variety of epitope peptides for specific TCR recognition, which is a key signal to start CTL (cytotoxic T lymphocyte) immunity (1). The MHC-I molecule presents the peptide through the closed peptide binding groove, and both ends of the peptide are anchored in the groove, so the length of the peptide is limited, generally 8-10 amino acids (2, 3). Long peptides (up to 15 peptides) can also be presented by MHC-I and cause a CTL response (4–6), but the ratio is relatively low. Long peptides often show a ‘bulge’ conformation, so both ends can be fixed in the peptide binding groove of MHC-I (7, 8). The conformational differences of peptides with different lengths will further affect the recognition of the TCR repertoire (6). However, some recent studies found that some long peptides can extend from the N- or C- end of the peptide binding groove, which reveals a new pattern of MHC-I presenting long peptides (9–13). However, compared with short peptides, the number of identified long peptides is much lower, which limits the in-depth study of the mechanism of MHC-I presenting long peptides. In the PDB library (https://www.rcsb.org/), the number of MHC-I complexes with short peptides is approximately 10 times that of long peptide complexes.

In recent years, the peptide length preference of MHC-I and its influence on the immune response have attracted increasing attention (5, 14, 15). The development of peptidomics provides powerful tools for accurately identifying the peptide binding characteristics of MHC-I, including peptide length preference (4, 13, 16–19). Mass spectrometry (MS) analysis of the human MHC-I (HLA-I) peptide ligand shows that some HLA-I alleles prefer to present longer peptides (5, 14, 15, 20). This indicates that the polymorphism of HLA-I can influence the length distribution of the presented peptides, but the mechanism behind it is still unclear.

Compared with humans and mice, there are fewer studies on the presentation of long peptides by MHC-I in other species. At present, only the complex structures of chicken and horse MHC-I and long peptides have been resolved (21, 22). However, due to the lack of high affinity monoclonal antibodies, cell lines and MHC-I haplotype experimental animals, the peptide length distributions of animal MHC-I revealed by cellular ligandome are relatively rare (9, 11, 22–27). Database-independent *de novo* peptide sequencing by MS can identify peptidomes without any database (28–30). We developed a new method *in vitro* to identify the peptide ligands of MHC-I through a random peptide library combined with LC-MS/MS and *de novo* sequencing (RPLD-MS) (17, 19). The random peptide library was refolded with MHC-I heavy chain and beta-2-microglobulin (β2-M) to form the peptide/MHC-I complex (pMHC-I). Then, pMHC-I was purified, and the bound peptides were eluted and sequenced by LC-MS/MS and *de novo* sequencing. Finally, the peptide-binding motif of MHC-I was determined. To date, the peptide binding properties of MHC-I molecules of five species, pig, bat, frog, lizard and shark, have been identified by RPLD-MS (17, 19, 31–33). This overcomes the limitations of laboratory animals, cell lines and antibodies, and lowers the threshold for the study of animal MHC-I.

Our studies on two highly homologous pig MHC-I (SLA-I) molecules, SLA-I*04:01 and SLA-1*13:01, showed that RPLD-MS can sensitively reflect the changes in peptide presentation caused by micropolymorphism. A key amino acid variation, Y99 in SLA-I*04:01 and F99 in SLA-1*13:01, makes SLA-1*04:01 present more nonapeptides than SLA-1*13:01. RPLD-MS can identify the length distribution of peptides presented by MHC-I *in vitro*, and it can focus on the influence of the polymorphism of MHC-I molecules, without considering the processes of peptide presentation *in vivo* (19). In addition, we also found a dodecapeptide RVEDVTNTAEYW (RW12 for short) which can bind to SLA-1*04:01. The complex structure (pSLA-1*04:01_RW12_) indicated that the first Arg of RW12 can extend from the N terminus of the peptide binding groove of SLA-1*04:01 (13). In this study, the length distributions of peptides presented by SLA-I*04:01 and SLA-1*13:01 were compared by RPLD-MS, and it was found that SLA-1*13:01 can bind more long peptides than SLA-1*04:01. Structural analysis revealed that SLA-1*04:01 and SLA-1*13:01 present RW12 in a markedly different manner, which is related to their different preferences for binding peptide lengths. This study will help us better understand how highly polymorphic MHC-I presents peptides of different lengths.

## Materials and Methods

### Synthesis of Random Peptide Library and Peptides

A random peptide repertoire was synthesized with a distribution of 19 amino acids other than cysteine at an equal molar ratio in every position, as previously reported (13, 29, 30). In brief, random peptide libraries of different lengths were synthesized separately according to this method and mixed in an equimolar ratio according to the average molecular mass before use.

Peptides with defined sequences used in this study, such as RVEDVTNTAEYW, were synthesized and purified to 99% by reverse-phase high performance liquid chromatography (HPLC) and mass spectrometry (SciLight Biotechnology).

All random peptide repertoires and peptides were stored in lyophilized aliquots at -80°C after synthesis and dissolved in dimethyl sulfoxide (DMSO) before use.

### Protein Preparation and Assembly

The DNA fragments encoding extracellular domains of SLA-1*13:01 (GenBank accession No. AB847437.1) and sβ2-M (GenBank accession No. BAG32341) were cloned into pET-21a (+) vectors (Novagen) and expressed as inclusion bodies in *E. coli* BL21 (DE3). The mutants at positions 99 of SLA-1*13:01 were cloned by overlap PCR and expressed as inclusion bodies in *E. coli* BL21 (DE3). The recombinant proteins were purified as described previously and dissolved in 6 M Gua-HCl (34).

The proteins were refolded by stepwise dilution using buffer containing 100 mM Tris-HCl (pH 8.0), 2 mM EDTA, 400 mM L-Arg HCl, 5 mM GSH, and 0.5 mM GSSH, and treated at 277 K for 12 hours. Before renaturation, a random peptide library containing 5.12 mg octapeptide, 5.76 mg nonapeptide, 6.4 mg decapeptide, 7.04 mg undecapeptide, 7.68 mg dodecapeptide, 8.32 mg thirteen peptide, 8.96 mg tetradecepeptide and 9.6 mg pentadecapeptide, or 10 mg of fixed peptide was dissolved and added to 1 L of refolding solution. Then, the solutions dissolved SLA-1 and sβ2-M inclusion bodies were individually added to refolding buffer at a 1:1 molar ratio. The refolded complexes were concentrated and purified with a Superdex 200 16/60 column in Tris buffer (20 mM Tris [pH 8] and 50 mM NaCl), followed by Resource Q anion-exchange chromatography (GE Healthcare) in Tris buffers (Buffer A: 20 mM Tris [pH 8] and 5 mM NaCl; Buffer B: 20 mM Tris [pH 8] and 500 mM NaCl).

### Isolation and Identification of High Affinity Peptides

The peptide-containing fraction in the complex was eluted according to a previous method (13, 19). In brief, the complex was treated with 10% acetic acid at 65°C for 15 minutes and the peptides were collected through a 3-kDa filter. Then, the peptide-containing fraction was desalted using a C18 tip and separated on the EasyNano LC 1000 system (San Jose, California, Thermo Fisher Scientific). Methods as follows: the peptide component was loaded into a trap column (5-μm pore size, 150-μm i.d. ×3-cm length, 100 Å) and separated by a custom C18 column (3-μm pore size, 75-μm i.d. 315-cm length, 100 Å), with a flow rate of 450 μL/minute. The 60-minute linear gradient was operated as follows: 3% B (0.1% formic acid in acetonitrile [v/v])/97% A (0.1% formic acid in H_2_O [v/v]) to 6% B in 8 min, 6% B to 22% B in 37 min, 22% B to 35% B in 8 min, 35% B to 100% B in 2 min, and 100% B for 5 min. The MS data were acquired by a Q Exactive HF (Bremen, Thermo Fisher Scientific) in data-dependent acquisition mode. The top 20 precursors by intensity in the mass range m/z 300 to 1800 were sequentially fragmented with higher-energy collisional dissociation and a normalized collision energy of 27. The dynamic exclusion time was 20 s. The automatic gain control for MS1 and MS2 was set to 3e6 and 1e, and the resolution for MS1 and MS2 was set to 120 and 30K.

Based on MS/MS information, peptides were resolved from each spectrum (FDR=1%) by *de novo* sequencing in Peaks Studio software. The parameters were set as follows: the enzyme was set to nonspecific, the variable modifications were adjusted oxidation (M)/deamidated (N,Q), the peptide mass tolerance was approximately ± 10 ppm, and the fragment mass tolerance was set to 0.02 Da. The identified peptides adjusted by the detection threshold (score ≥ 50) are listed in **Table S1**.

To determine the RXEDVTNTAEYW (X represents any of twenty amino acids) library, MaxQuant1.6 software was used to search peptides based on MS/MS information. Posttranslational modifications (PTMs) were set to false, and the MS/MS match tolerance was set to 20 ppm. No-specificity was selected in the digestion option. The protein FDR was set to 0.01.

### Thermal Stabilities of the pSLA-1 Molecules

The circular dichroism (CD) spectra of the peptide-SLA-1 (pSLA-1) complexes were measured on a CD instrument (Chirascan; Applied Photophysic, Ltd.) using a Jasco J-810 spectrometer equipped with a water-circulating cell holder. As the temperature rose from 20°C to 80°C at a rate of 1°C/minute. A 1-mm optical path length cell was used for monitoring at 218 nm. The instrument used a thermistor to detect the temperature of the solution. Finally, the ratio of unfolded protein to the mean residue ellipticity (*θ*) was calculated. The unfolded score is shown as (θ *- θ_N_)*/(*θ_U_ - θ_N_*), where *θ_N_* and *θ_U_* are the mean residual ellipticity values in fully folded and fully unfolded states, respectively. The midpoint transition temperature (*Tm*) is calculated from the denaturation curve data in the Origin 9.1 program (OriginLab).

### Crystallization and Data Collection

The refolded and purified protein complex was concentrated and performed using the sitting-drop and hanging-drop vapor diffusion method at 277 K and 291K. Complex crystals of SLA-1*13:01 with peptide RVEDVTNTAEYW (pSLA-1*13:01_RW12_) were obtained under PEG/Ion solution No.3 (0.2 M ammonium fluoride, 20% w/v polyethylene glycol 3,350). Prior to X-ray diffraction, the crystals were immersed in a stock solution containing 17% glycerol as a cryoprotectant, and then flash-cooled in a 100 K gaseous nitrogen stream. Then, the diffraction data of pSLA-1*13:01_RW12_ were collected and the R-AXIS IV^++^ imaging plate detector was used to resolve the resolution of 2.5 Å on Beamline BL19U1 (wavelength, 0.97892 Å) of the Shanghai Synchrotron Radiation Facility (Shanghai, China). The HKL-3000 software package (HKL Research) was used to index, integrate, scale and merge data (35). The crystallographic statistics for the complexes are listed in **Table 1**.

**Table 1:** X-ray diffraction data processing and refinement statistics.

Parameter	pSLA-1*13:01_RW12_
**Data collection**	
Space group	P12_1_1
Unit cell parameters (Å)	95.17, 44.24, 199.63 90.00, 90.03, 90.00
Resolution range (Å)	99.81-2.16 (2.28-2.16)
Total reflections	482,111
Unique reflections	87,860
*R*_merge_ (%) *^b^*	17.6 (32.6)
Avg *I*/σ(*I*)	6.0 (3.7)
Completeness (%)	97.1
Redundancy	5.5 (5.1)
**Refinement**	
Resolution (Å)	30.00-2.50
No.reflections	55,584
*R*_factor_ (%) *^c^*	24.67
*R*_free_ (%)	28.52
R M S.Deviations	
Bonds (Å)	0.007
Angles (°)	1.254
Average B factor	44.530
Ramachandran plot quality	
Most favored region (%)	94.02
Allowed region (%)	5.98
Disallowed region (%)	0.00

a Values in parentheses are for the highest-resolution shell.

b Rmerge = Σ_hkl_Σ_i_|I_i_(hkl) – 〈I(hkl)〉|/Σ_hkl_Σ_i_ I_i_(hkl), where I_i_(hkl) is the observed intensity and 〈I(hkl)〉is the average intensity from multiple measurements.

c R=Σ_hkl_|| F_obs_ | – k | Fcalc | |Σ_hkl_| F_obs_|, where R_free_ is calculated for a randomly chosen 5% of reflections and R_work_ is calculated for the remaining 95% of reflections used for structure refinement.

### Structural Determination and Analysis

Using SLA-1*13:01 (Protein Data Bank code 6KWO) as a search model, the structure of the pSLA-1*13:01_RW12_ complex was determined by molecular replacement using the Phaser program (36). The model was rebuilt using COOT (37) and refined by REFMACS5 (38). Refinement rounds were implemented using the refinement program in the PHENIX package with isotropic atomic displacement parameter (ADP) refinement and bulk solvent modeling (39). Finally, the stereochemical quality of the final model was assessed using the PROCHECK program (40). Structural illustrations and electron density-related figures were drawn using PyMOL software. Multiple-sequence alignment was performed with Clustal Omega (http://www.ebi.ac.uk/Tools/msa/clustalo/). The accessible surface area (ASA) and buried surface area (BSA) were calculated with PDB in Europe Proteins, Interfaces, Structures and Assemblies (PDBePISA, http://www.ebi.ac.uk/msd-srv/prot_int/pistart.html), and the B factor was calculated with CCP4.

## Results

### SLA-1*13:01 Binds More Long Peptides *In Vitro* Than SLA-1*04:01

**Figure 1:**
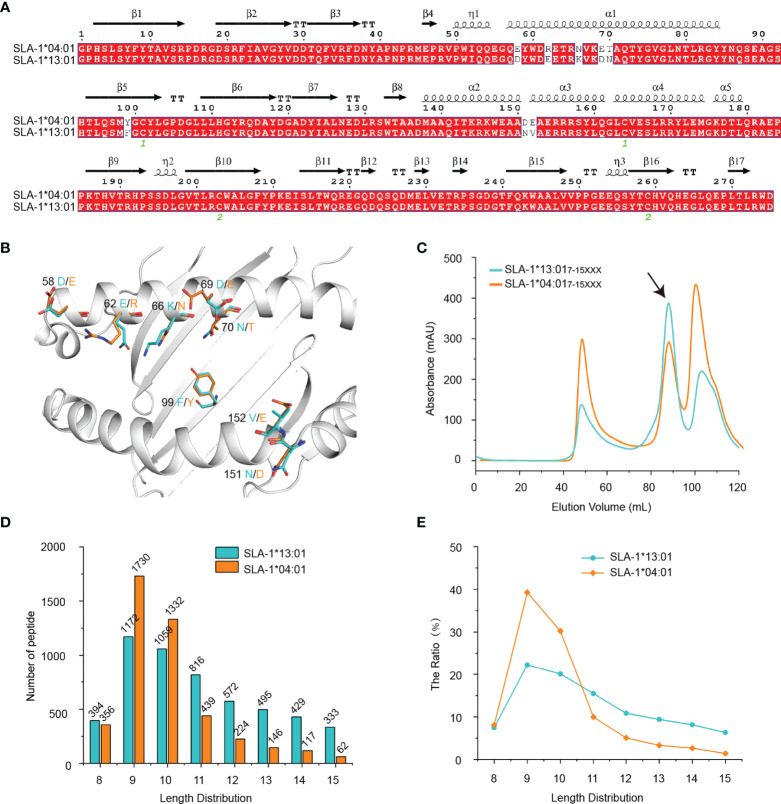
Analysis of the refolding of SLA-1*13:01 and SLA-1*04:01 bound peptide libraries and the determination of the eluted peptide length and quantity. **(A)** Structure-based sequence alignment of SLA-1*04:01 and SLA-1*13:01. **(B)** The structural location of the different residues between SLA-1*13:01 and SLA-1*04:01. **(C)** Gel filtration chromatograms of the *in vitro* refolding test of SLA-1*13:01 and SLA-1*04:01 with random peptides of mixed length. The black arrows point to the peak of the compound. **(D, E)** The length and quantity comparison between SLA-1*13:01 and SLA-1*04:01 eluted peptides.

SLA-1*13:01 differs from SLA-1*04:01 by only eight residues, and they are all distributed on the peptide binding platform composed of α1 and α2 domains (**Figures 1A, B**). Our previous research showed that SLA-1*04:01 binds much more nonapeptides than SLA-1*13:01 (19). However, the *in vitro* refolding results of SLA-1*04:01 and SLA-1*13:01 with a mixed random peptide library of 7-15 amino acids showed that the peak of pSLA-1*13:01 is higher than the peak of pSLA-1*04:01, indicating SLA-1*13:01 can bind more random peptides of mixed length than SLA-1*04:01 (**Figure 1C**). RPLD-MS showed that compared with SLA-1*04:01 (13), SLA-1*13:01 could bind fewer short peptides of 8-10 amino acids, but more long peptides of 11-15 amino acids (**Figure 1D** and **Table S1**). It should be pointed out that among the peptides bound by SLA-1*13:01 and SLA-1*04:01, nonapeptide and decapeptide are the main peptides, which agrees with the consensus on the length distribution of MHC-I binding peptides. Compared with SLA-1*04:01, SLA-1*13:01 has a lower amount and proportion of binding nonapeptides (**Figures 1D, E**), which consists of the previous data (19).

### Different Conformations of Dodecapeptide RW12 Bound by SLA-1*13:01 and SLA-1*04:01

**Figure 2:**
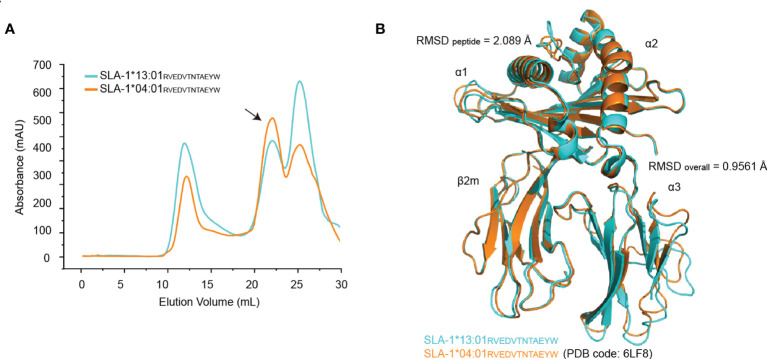
Structural determination of the pSLA-1*13:01_RVEDVTNTAEYW_ complex. **(A)** Visual display of SLA-1*13:01 and SLA-1*04:01 *in vitro* refolding efficiency with the peptide RVEDVTNTAEYW by gel filtration chromatograms. The black arrows point to the peak of the compound. **(B)** The overall structural comparison between pSLA-1*13:01_RW12_ (cyan) and pSLA-1*04:01_RW12_ (orange) presented in cartoon form.

Structural analysis of SLA-1*13:01 and SLA-1*04:01 complexed with long peptides is helpful to clarify their different preferences for peptide length. We have previously identified a dodecapeptide (RW12) that can bind to SLA-1*04:01 and solved the structure of their complex pSLA-1*04:01_RW12_ (13). Here, an *in vitro* refolding experiment showed that RW12 can also combine with SLA-1*13:01 to form the stable complex pSLA-1*13:01_RW12_ (**Figure 2A**). After purification and crystal screening of pSLA-1*13:01_RW12_, we successfully obtained the crystal and finally solved its structure at 2.5 Å, the same resolution of pSLA-1*04:01_RW12_ (**Table 1**). Structural analysis confirms that bound peptide conformation is not affected by crystal-crystal packing. The structural comparison between pSLA-1*04:01_RW12_ and pSLA-1*13:01_RW12_ showed that their overall structural similarity was high (RMSD = 0.9561), but the conformations of the presented RW12 peptide were obviously different (RMSD = 2.089) (**Figure 2B**).

**Figure 3:**
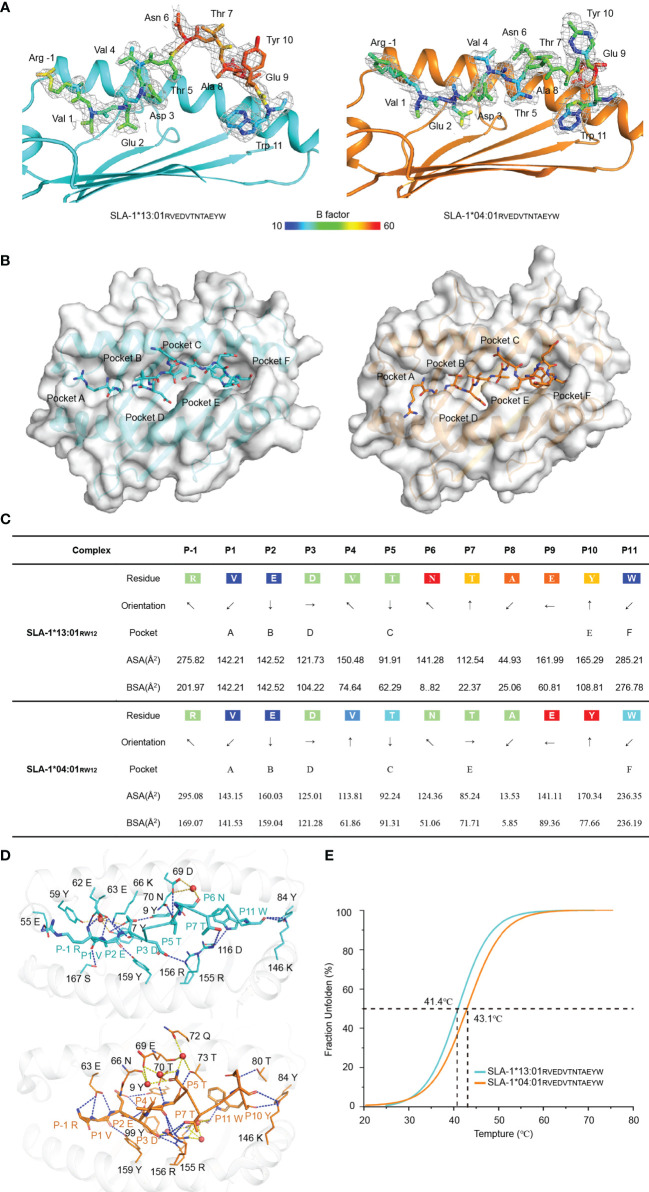
Electron density and overall conformation of the structurally defined peptides. **(A)** Electron densities and overall conformations of the peptide RVEDVTNTAEYW from the solved pSLA-1*13:01_RW12_ and pSLA-1*04:01_RW12_ complexes. Simulated CNS annealing omit maps calculated for the peptides are shown in blue at a contour of 1.0. **(B)** The structural location of the peptide RVEDVTNTAEYW residues inserted into the pocket. **(C)** General side chain orientations and the different interfacing areas of peptides presented in a table, as viewed in profile from the peptide N-terminus toward the C-terminus. Black arrows indicate the directions in which the residues point: up is toward the TCR, down is toward the floor of the ABG, left is toward the α1 helix domain, and right is toward the α2 helix domain. Pockets accommodating each residue are listed under the corresponding anchors within the ABG. ASA, accessible surface area of each residue; BSA, buried surface area of the residues. **(D)** Comparison of the forces that mediate peptide stabilization between pSLA-1*13:01_RW12_ and pSLA-1*04:01_RW12_. The red sphere is the solvent molecule. **(E)** Thermal stabilities of pSLA-1*13:01_RW12_ and pSLA-1*04:01_RW12_ analyzed by the CD spectroscopy. The stabilities can be measured by the *Tm* value. The *Tm* values of the complexes are labeled.

In the structures of pSLA-1*04:01_RW12_ and pSLA-1*13:01_RW12_, the electronic density maps of the RW12 peptide are clear and can be used for credible comparative analysis (**Figure 3A**). Our previous study showed that the binding mode of RW12 is different from the classical MHC-I peptide binding mode. The first residue Arg (P-1-R) at the N terminus of RW12 extends out of the peptide binding groove of SLA-1*04:01, the second residue Val (P1-V) is turned over and its side chain is accommodated by the A pocket (13). The same is true of the interaction between the N terminus of RW12 and SLA-1*13:01 (**Figure 3B**). Except for the N terminus, the conformations of the rest of RW12 are significantly different in SLA-1*13:01 and SLA-1*04:01. RW12 obviously protrudes from the peptide binding groove of SLA-1*13:01 but is deeply embedded in the groove of SLA-1*04:01 (**Figure 3B**), and the side chain orientation of each residue is also different (**Figure 3C**).

The hydrogen bonds between RW12 and SLA-1*13:01 are significantly less than that between RW12 and SLA-1*04:01, including the hydrogen bonds mediated by water molecules, especially in the middle region of the peptide binding groove (**Figure 3D** and **Table 2**). Compared with pSLA-1*04:01_RW12_, the residues from P6 to P10 of RW12 in pSLA-1*13:01_RW12_ have higher B factors, which indicates that these residues protruding from the peptide binding groove are less constrained (**Figure 3A**). The results of circular dichroism showed that the thermal stability of pSLA-1*04:01_RW12_ is slightly higher than that of pSLA-1*13:01_RW12_, consisting with the numbers of hydrogen bonds with RW12, although both of them can keep stable and crystallize (**Figure 3E**).

**Table 2:** The interactions between the peptide and the PBG of pSLA-1*13:01_RW12_.

Complex	Hydrogen bonds and salt bridges				van der Waals contactresidues
	Peptide		Heavy Chain		
	Residue	Atom	Residue	Atom	
pSLA-1*13:01_RW12_	P-1-Arg	NH1	Glu^55^	OE2	Glu^55^, Tyr^59^, Glu^62^, Glu^63^, Leu^163^, Glu^166^, Ser^167^, Arg^170^, Tyr^171^
		N	Glu^62^	OE2	
			Glu^63^	OE1	
		O	Ser^167^	OG	
	P1-Val	N	Glu^63^	OE2	Leu^5^, Tyr^7^, Phe^33^, Tyr^59^, Glu^63^, Tyr^159^, Leu^163^, Ser^167^, Tyr^171^
		O	Tyr^159^	OH	
	P2-Glu	OE1	Tyr^7^	OH	Tyr^7^, Tyr^9^, Met^45^, Glu^63^, Lys^66^, Val^67^, Asn^70^, Phe^99^, Tyr^159^
		OE2	Tyr^9^	OH	
		N	Glu^63^	OE2	
		O	Lys^66^	NZ	
	P3-Asp	OD2	Arg^156^	NE	Asn^70^, Phe^99^, Arg^114^, Arg^156^, Tyr^159^
	P4-Val				Arg^65^, Lys^66^
	P5-Thr	OG1	Asp^69^	OD1	Asp^69^, Asn^70^, Arg^155^, Arg^156^
			Asn^70^	OD2	
	P6-Asn				Arg^65^, Asp^69^
	P7-Thr	OG1	Arg^155^	NH1	Arg^155^, Arg^156^
	P8-Ala				Arg^156^
	P9-Glu				Thr^73^, Val^76^, Gly^77^, Thr^80^
	P10-Tyr				Thr^143^, Lys^146^, Trp^147^, Ala^150^, Val^152^
	P11-Trp	O	Tyr^84^	OH	Thr^73^, Tyr^74^, Gly^77^, Thr^80^, Lue^81^, Tyr^84^, Leu^95^, Ser^97^, Arg^114^, Asp^116^, Thr^143^, Lys^146^, Trp^147^
			Lys^146^	NZ	
		NE1	Asp^116^	OD2	

### Effects of Different Amino Acids in SLA-1*13:01 and SLA-1*04:01 on the RW12 Conformation

**Figure 4:**
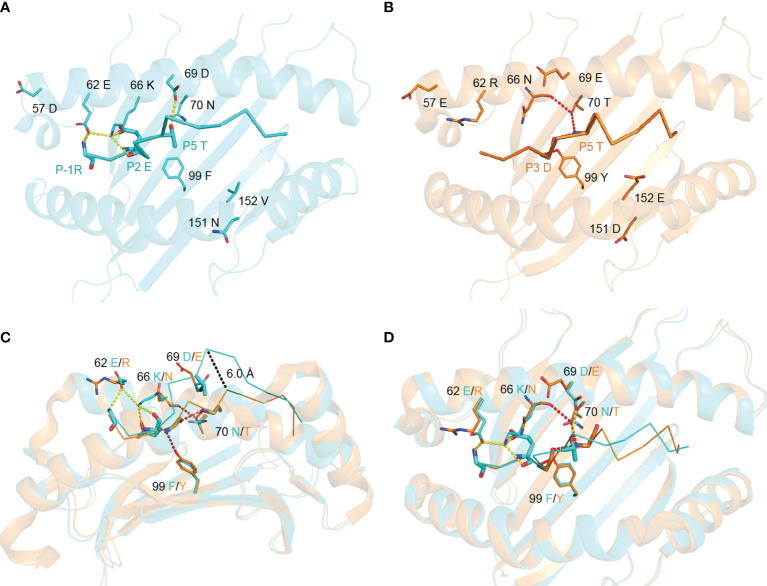
Contributions of variant amino acids between SLA-1*13:01 and SLA-1*04:01 to the conformation of peptide RVEDVTNTAEYW. **(A)** Analysis of the interaction between different amino acids and peptide in pSLA-1*13:01_RW12_. **(B)** Analysis of the interaction between variant amino acids and peptide in pSLA-1*04:01 _RW12_. **(C, D)** Comparison of the effect of amino acid differences between SLA-1*13:01 and SLA-1*04:01 on peptide conformation.

The conformational difference between RW12 presented by SLA-1*13:01 and SLA-1*04:01 is caused by the different amino acids between these two SLA-I alleles. Structural analysis showed that only five of the eight differential amino acids directly interact with RW12, and the variable residues at positions 57, 151 and 152 do not contact RW12. 62E, 66K and 69D in SLA-1*13:01 can form hydrogen bonds with P-1-R, P2-E and P5-T of RW12 (**Figure 4A**), and 70T and 99Y in SLA-1*04:01 can form hydrogen bonds with P3-D and P5-T of RW12 (**Figure 4B**).

62E/R and 66K/N at the N terminus of the peptide binding groove did significantly alter the conformation of the P-1 to P2 residues of RW12 (**Figures 4C, D**). The conformational difference of RW12 starts from P3-D, the P3-D position in pSLA-1*13:01_RW12_ is relatively higher (**Figure 4C**), the positions of the subsequent residues are more elevated, and their side chain orientations are more variable (**Figure 3C**). These results indicated that the difference between SLA-1*04:01 and SLA-1*13:01 in presenting RW12 peptide may start from the interaction with P3-D and be consolidated later, while 69D/E, 70N/T and 99F/Y are just located in this region (**Figures 4C, D**).

**Figure 5:**
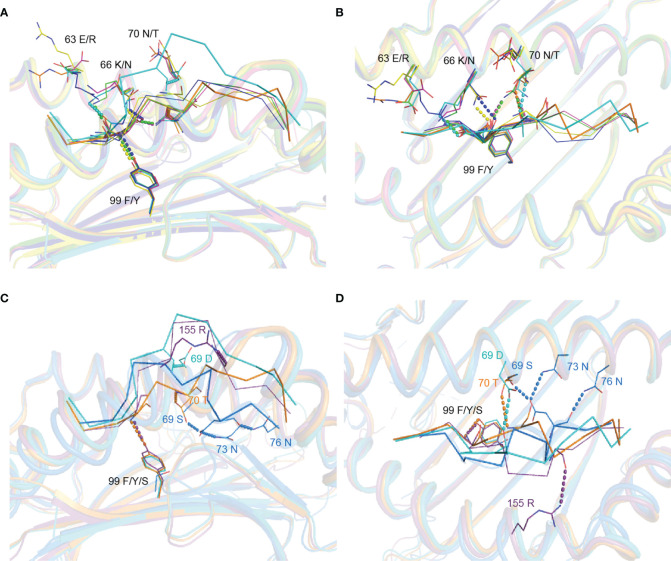
Analysis of the effect of variant residues between SLA-1*13:01 and SLA-1*04:01 on MHC-I binding peptides. **(A, B)** Structural comparison between determined pSLA-1*13:01_RW12_ (cyan), pSLA-1*04:01_RW12_ (orange, PDB code 6LF8), pSLA-1*04:01_MY9_ (blue, PDB code 6KWK), pSLA-1*13:01(F99Y)_NW9_ (green, PDB code 6KWN) and pSLA-1*13:01_EW9_ (magenta, PDB code 6KWO). The dotted lines of different colors indicate the interaction between the peptides and the residues of the antigen binding groove in different structures. **(C, D)** Structural comparison between determined pSLA-1*13:01_RW12_ (cyan), pSLA-1*04:01_RW12_ (orange, PDB code 6LF8), pMamu*B17_IW11_ (purple, PDB code 3RWD) and pBF2*21:01_GL11_ (purple blue, PDB code 2YF1). The dotted lines of different colors indicate the interaction between the peptides and the residues of the antigen binding groove in different structures.

Our previous study showed that 99Y/F is the key factor that leads to a significant difference between SLA-1*04:01 and SLA-1*13:01 when binding nonapeptides (19). 99Y in SLA-1*04:01 can form a hydrogen bond with the main chain of the P3 residue, but in SLA-1*13:01, 99F cannot form a similar hydrogen bond and leads to a decrease in its ability to bind nonapeptides. If the 99Y/F of SLA-1*04:01 and SLA-1*13:01 are replaced by mutations, the ability to bind nonapeptides between them will also be exchanged. Similar to the binding of nonapeptides, the main chain of P3-D of RW12 forms a hydrogen bond with 99Y of SLA-1*04:01, but not with 99F of SLA-1*13:01. The conformational difference of RW12 also starts from P3-D, 99Y of SLA-1*04:01 makes P3-D of RW12 tied to the bottom of the groove but 99F of SLA-1*13:01 cannot (**Figure 4**). To further analyze the role of 99Y/F, we compared the other solved structures of SLA-1*04:01, SLA-1*13:01 and its mutant SLA-1*13:01(F99Y). 99Y can form a conserved hydrogen bond with the P3 main chain in all structures (**Figure 5A**), so we believe that 99Y/F has an important influence on peptide binding, regardless of peptide length.

Although the other variant residues do not interact with peptides in the stable way (**Figures 5A, B**), but they also have their own unique role in influencing peptide conformation. 70T of SLA-1*04:01forms a hydrogen bond with the main chain of P5-T, further limiting the subsequent residues of RW12 in the peptide binding groove (**Figures 4B–D**). 69D of SLA-1*13:01 forms a hydrogen bond with P5-T, which fixes the middle of RW12 outside the peptide binding groove, jointly determine the conformation of RW12 protruding out of the SLA-I*13:01 groove (**Figures 4A, C, D**). Overall, RW12 presents a typical ‘bulge’ conformation in pSLA-1*13:01_RW12_, while it is embedded in the groove in pSLA-1*04:01_RW12_. The maximum position deviation between them can reach 6.0 Å (**Figure 4C**).

By comparing the structures of other resolved MHC-I and undecapeptide complexes, we also found that the 99^th^ residue cannot independently determine the conformation of the peptide. For example, 99S of the chicken MHC-I molecule (BF2*2101) does not form hydrogen bonds with the P3 residue, similar to SLA-1*13:01 and RW12, but its 69S, 73N and 76N residues are bound to peptide, which limits the whole peptide in the groove, similar to SLA-1*04:01 and RW12. 99Y of the monkey MHC-I molecule (Mamu*B17) forms a hydrogen bond with the P3 residue, similar to SLA-1*04:01 and RW12, but there is no strong restriction on the peptide, similar to SLA-1*13:01 and RW12 (**Figures 5C, D**). Therefore, for SLA-1*04:01 and SLA-1*13:01, the difference of 99Y/F should be critical factor for the binding of peptide, but it needs to cooperate with other different amino acids to jointly determine the conformation of peptide.

### The N-terminal Extended Peptide Presentation Mode Has No Obvious Effect on the Difference of Peptide Length Distribution Bound by SLA-1*13:01 and SLA-1*04:01

The N terminus of RW12 can extend out of the peptide binding groove of SLA-1*04:01 and SLA-1*13:01, while 62E and 66K in SLA-1*13:01 can form hydrogen bonds with P-1-R and P2-E (**Figure 4A**). This may result in SLA-1*13:01 having a stronger ability to present N-terminal extended peptides than SLA-1*04:01, thus affecting their preference for long peptides. Therefore, it is necessary to compare the capabilities of SLA-1*04:01 and SLA-1*13:01 in presenting N-terminal extended peptides.

**Figure 6:**
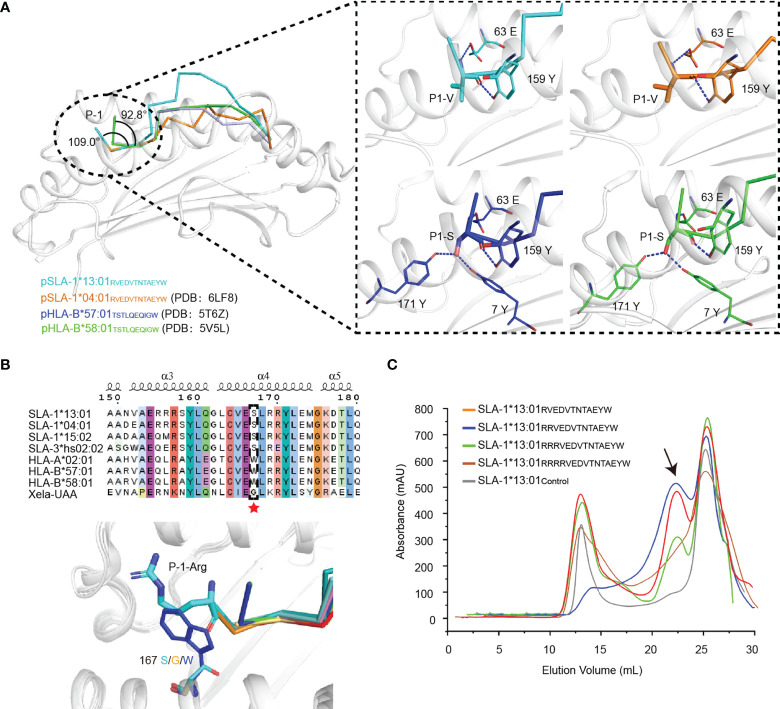
The peptide N-terminal extension mode of the MHC-I family. **(A)** The consistent mode of the N-terminally extended peptide presented by MHC-I. Comparison of the forces between the A pocket and peptides in SLA-I and HLA-I. **(B)** Sequence alignment and structural comparison of residue at position 167 in SLA-I, HLA-I and Xela-UAA. The amino acids at position 167 are highlighted by a red pentacle. **(C)** The *in vitro* refolding efficiency of SLA-1*13:01 was measured with peptides with different numbers of arginines added to the N-terminus of RW12. The black arrow indicates the peak of the complex.

At present, there are only four pMHC-I complexes with N-terminal extending peptides, pSLA-1*04:01_RW12_, pSLA-1*13:01_RW12_, pHLA-B*57:01_TSTLQEQIGW_ (pHLA-B*57:01_TW10_) and pHLA-B*58:01_TW10_ (9, 12, 13). SLA-I and HLA-I have similar patterns of binding N-terminal extension peptide, but they also clearly show distinguishable species characteristics (**Figure 6A**). The change in the binding mode of the P1 residue of the peptide is the key to the extension of the peptide N terminus. Compared with other peptides in classical presentation mode, the P1 residues of RW12 and TW10 are reversed, and their side chains are inserted into the A pocket, which leads to the elevation of the Cα position of the P1 residue. Then, the main chain N atom and O atom of the P1 residue form hydrogen bonds with 63E on the α1 helix and 159Y on the α2 helix, respectively. The 63^rd^ residue is polymorphic, and our previous mutation studies show that 63E is necessary for this binding pattern (13). The angle between P-1 and P1 residues in RW12 is obviously larger than that in TW10 (**Figure 6B**) because the species characteristic of 167S in SLA-I alleles makes the N terminus of the peptide binding groove of SLA-I open and is beneficial to the extension of the P-1 residue (13).

Although 62E and 66K in SLA-1*13:01 can form hydrogen bonds with P-1-R and P2-E, there is no strong interaction with the P1 residue (**Figure 4A**), nor does it change the RW12 N-terminal extension (**Figure 6**). By adding excess residues at the N terminus, we previously proved that SLA-I * 04:01 can allow the extension of up to three residues (13). SLA-1*13:01 could only tolerate the extension of 1-3 residues from the N terminus of RW12 (**Figure 6C**). Therefore, we believe that SLA-1*13:01 does not have a stronger binding ability for the N-terminal extended peptide than SLA-I*04:01, and the N-terminal extended peptide presentation mode has no obvious influence on the difference in peptide length preferences between SLA-1*13:01 and SLA-1*04:01.

## Discussion

The length of the peptide can affect the recognition of MHC-I restricted epitopes by TCR, so the identification of T cell epitopes should consider the length of epitopes (6). Different HLA-I alleles have various preferences for the length of binding peptides (5). However, naturally presented peptides are not only influenced by MHC allele-specific binding preference, but also depend on many processes such as digestion and transportation of peptides in cells (41–45). Therefore, there are certain limitations in studying the peptide length preference of MHC-I alleles by MHC-I ligandomes *in vivo*, which further affects the prediction, screening and identification of long peptide epitopes.

RPLD-MS can determine the MHC-I random peptide ligandome *in vitro* and quickly identify MHC-I restricted peptide epitopes, which is especially suitable for studying unknown animal MHC-I molecules lacking research background and conditions (17, 19, 31–33). Our previous studies proved that RPLD-MS can sensitively reflect the different nonapeptide binding capabilities *in vitro* between SLA-1*04:01 and SLA-1*13:01 caused by a single residue variation (99Y/F) (19). In this study, we quantitatively mapped the length distribution of SLA-1*04:01 and SLA-1*13:01 ligand peptidomes with RPLD-MS. These results showed that RPLD-MS is suitable for studying the effect of polymorphisms on peptide length preferences of MHC-I alleles: first, it eliminates the interference of peptide processing on the peptide length distribution *in vivo*, and focuses on the binding preference of the MHC-I molecule itself; second, its sensitivity and accuracy are enough to reflect the influence of micropolymorphism on peptide length preference. Ultimately, of course, identified natural presented peptidomes will be required to determine whether the preference for peptide length by MHC-I itself is sufficiently pronounced.

Compared with SLA-1*04:01, why is SLA-1*13:01 weak in binding short peptides but strong in binding long peptides? This is due to the difference in amino acids between them, which leads to different binding ways of short and long peptides. For short peptides, regardless of SLA-1*04:01 or SLA-I*13:01, the whole peptide should be accommodated in the peptide binding groove to form enough affinity with limited length. The differential amino acids of SLA-1*04:01 and SLA-1*13:01 are not located in the pockets that restrict the side chains of peptides, so the short peptides that they can bind to have similar motifs. However, 99F leads to the lack of hydrogen bond between SLA-1*13:01 and the mainchain of P3 residue, which plays a key role in stabilizing peptide binding. Therefore, only short peptides that strongly bind to SLA-1*04:01 can bear the loss of affinity caused by 99F and stably bind to SLA-1*13:01. This caused the capability of SLA-1*13:01 to bind short peptides to be weaker than SLA-1*04:01 (19). For the binding of long peptides with more than 10 amino acids, these two SLA-I molecules are different. 99Y and 70T of SLA-1*04:01 are bound to the main chain of RW12, which limits RW12 in the peptide binding groove. This binding does not involve the sidechain of the peptide, indicating that SLA-1*04:01 should bind other long peptides in a similar way. The whole long peptide needs to be completely contained by its groove, so SLA-1*04:01 is restrictive for long peptides. However, 99F and 70N of SLA-1*13:01 have no restriction on long peptides, and long peptides can protrude out of the binding groove if they can bind stably. Therefore, SLA-1*13:01 reduces the restriction on long peptides and increases the types and quantities of long peptides that may be bound.

It is a cross-species phenomenon that both ends of the peptide extend from the peptide binding groove of MHC-I (9–13). At present, extension of the N terminus of peptides has been found in HLA-I (HLA-B*57:01, HLA-B*58:01) and SLA-I (SLA-1*04:01, SLA-1*13:01), and all the residues contained in the A pocket have turned over in a similar way. The species characteristic of S167 opens the N terminus of the SLA-I peptide binding groove, which is helpful for the N-terminal extension of the peptide. However, both SLA-1*04:01 and SLA-1*13:01 can only bear 1-3 redundant N-terminal residues, so this novel peptide binding mode does not affect their preferences for peptide length.

At present, the identification of MHC-I restricted CTL epitopes focuses on short peptides with 8-10 amino acids and relatively ignores long peptide epitopes. However, there is increasing evidence that long peptide epitopes also play an important role in the CTL response (4–6). Our study showed that long peptides accounted for a considerable proportion of the SLA-I restricted epitopes. First, SLA-I species-specific S167 facilitates N-terminal nested long peptide binding; Second, some alleles, such as SLA-1*13:01, bind to a higher proportion of long peptides. Therefore, long peptides should not be ignored in the identification of SLA-I limiting epitopes. Because the sequences of SLA-1*04:01 and SLA-1*13:01 are highly homologous, it is helpful to understand how the MHC-I polymorphism affects peptide length preference by analyzing their peptide length distribution and basis. Further exploration of the relationship between SLA-I polymorphism and the length of binding peptide will help us to screen SLA-I restricted T cell epitopes more comprehensively, avoid the omission of long peptide epitopes, and develop effective epitope vaccines.

## Data Availability Statement

The coordinate and structure factor for the pSLA-1*13:01_RW12_ complex have been deposited in the Protein Data Bank (https://www.rcsb.org/) with the following accession number: 6LF9. The mass spectrometry proteomics data have been deposited to the ProteomeXchange Consortium *via* the PRIDE (https://www.ebi.ac.uk/pride/) partner repository with the dataset identifier PXD020818.

## Author Contributions

NZ and XW conceived and designed the study. XW and SL performed the experiments. SW and GF collected and analyzed the crystal data. XX and ZL analyzed the LC-MS/MS data. XW and NZ wrote and revised the manuscript. All authors contributed to the article and approved the submitted version.

## Funding

This work was supported financially by grants from the National Key Research and Development Program of China (2021YFD1800100), the National Natural Science Foundation of China (NSFC) (31830097, http://www.nsfc.gov.cn), the National Natural Science Foundation of China (NSFC) (31201887, http://www.nsfc.gov.cn), the Natural Science Foundation of Beijing Municipality (6182029, http://www.bjkw.gov.cn/), Major Science and Technology Project of Liaoning Province (2020JH1/10200003) and the 2115 Talent Development Program of China Agricultural University.

## Conflict of Interest

The authors declare that the research was conducted in the absence of any commercial or financial relationships that could be construed as a potential conflict of interest.

## Publisher’s Note

All claims expressed in this article are solely those of the authors and do not necessarily represent those of their affiliated organizations, or those of the publisher, the editors and the reviewers. Any product that may be evaluated in this article, or claim that may be made by its manufacturer, is not guaranteed or endorsed by the publisher.
